# Efficiency of hydrogen peroxide in improving disinfection of ICU rooms

**DOI:** 10.1186/s13054-015-0752-9

**Published:** 2015-02-02

**Authors:** Caroline Blazejewski, Frédéric Wallet, Anahita Rouzé, Rémi Le Guern, Sylvie Ponthieux, Julia Salleron, Saad Nseir

**Affiliations:** Critical Care Center, University Hospital of Lille, Rue E. Laine, 59037 Lille Cedex, France; Microbiology Department, University Hospital of Lille, boulevard du Pr. Leclercq, 59000 Lille Cedex, France; Statistics Department, University Hospital of Lille, 1 place de Verdun, 59037 Lille Cedex, France; Medicine School, Univeristy of Lille, 1 place de Verdun, 59037 Lille Cedex, France

## Abstract

**Introduction:**

The primary objective of this study was to determine the efficiency of hydrogen peroxide (H_2_O_2_) techniques in disinfection of ICU rooms contaminated with multidrug-resistant organisms (MDRO) after patient discharge. Secondary objectives included comparison of the efficiency of a vaporizator (HPV, Bioquell®) and an aerosolizer using H_2_O_2_, and peracetic acid (aHPP, Anios®) in MDRO environmental disinfection, and assessment of toxicity of these techniques.

**Methods:**

This prospective cross-over study was conducted in five medical and surgical ICUs located in one University hospital, during a 12-week period. Routine terminal cleaning was followed by H_2_O_2_ disinfection. A total of 24 environmental bacteriological samplings were collected per room, from eight frequently touched surfaces, at three time-points: after patient discharge (T0), after terminal cleaning (T1) and after H_2_O_2_ disinfection (T2).

**Results:**

In total 182 rooms were studied, including 89 (49%) disinfected with aHPP and 93 (51%) with HPV. At T0, 15/182 (8%) rooms were contaminated with at least 1 MDRO (extended spectrum β–lactamase-producing Gram-negative bacilli 50%, imipenem resistant *Acinetobacter baumannii* 29%, methicillin-resistant *Staphylococcus aureus* 17%, and *Pseudomonas aeruginosa* resistant to ceftazidime or imipenem 4%). Routine terminal cleaning reduced environmental bacterial load (*P* <0.001) without efficiency on MDRO (15/182 (8%) rooms at T0 versus 11/182 (6%) at T1; *P* = 0.371). H_2_O_2_ technologies were efficient for environmental MDRO decontamination (6% of rooms contaminated with MDRO at T1 versus 0.5% at T2, *P* = 0.004). Patient characteristics were similar in aHPP and HPV groups. No significant difference was found between aHPP and HPV regarding the rate of rooms contaminated with MDRO at T2 (*P* = 0.313). 42% of room occupants were MDRO carriers. The highest rate of rooms contaminated with MDRO was found in rooms where patients stayed for a longer period of time, and where a patient with MDRO was hospitalized. The residual concentration of H_2_O_2_ appears to be higher using aHPP, compared with HPV.

**Conclusions:**

H_2_O_2_ treatment is efficient in reducing MDRO contaminated rooms in the ICU. No significant difference was found between aHPP and HPV regarding their disinfection efficiency.

## Introduction

Intensive care unit (ICU)-acquired infection is a common adverse event in critically ill patients [1]. This infection is frequently related to multidrug-resistant organisms (MDRO), and is associated with high morbidity and mortality rates [2]. Infections related to MDRO are frequently associated with inappropriate initial antimicrobial treatment and an increased mortality rate [3]. Therefore, the prevention of ICU-acquired infections related to MDRO is a crucial issue.

The environment is a major reservoir for MDRO. These organisms remain viable on various inanimate surfaces for days to months [4,5]. Pathogens can then be transferred from the environment to patients directly by contact between patients and the contaminated environment and indirectly through healthcare workers’ (HCW) hands. Environmental persistence of pathogens is also thought to facilitate vertical transmission [6,7]. Admission to a room previously occupied by a patient colonized or infected with methicillin-resistant *Staphylococcus aureus* (MRSA), vancomycin-resistant enterococci (VRE), *Acinetobacter baumannii*, *Pseudomonas aeruginosa* or *Clostridium difficile* increases the risk of acquiring the same organism by the subsequent patient admitted in the same room [8-11].

Current every-day and terminal cleaning methods seem to be microbiologically ineffective [12]. This fact is generally under-recognized since environmental microbiological quality is rarely assessed. Hygiene failure is partly due to HCW understaffing or over-workload, hardly reachable surfaces, and ineffectiveness of common disinfectants against bacteria growing within biofilm. Therefore, new automated disinfection methods are being increasingly studied. Hydrogen peroxide (H_2_O_2_) generators are the most investigated, including H_2_O_2_ aerosolization (aHP), and H_2_O_2_ vaporization (HPV) [6].

Previous studies demonstrated *in vitro*, *in situ* and clinical effectiveness of H_2_O_2_ techniques in reducing environment contamination by MDRO [13-23]. However, several limitations of these studies should be taken into account, including the small number of studied ICU rooms, the absence of systematic environmental samples and the focus on specific MDRO or specific population. Further, to our knowledge, no study has compared the efficiency of an aerosolizer using H_2_O_2_ and peracetic acid (aHPP), and HPV techniques.

The primary objective of this study was to determine the efficiency of H_2_O_2_ techniques in disinfection of ICU rooms contaminated with MDRO after patient discharge. Secondary objectives included comparison of the efficiency of an HPV system (Bioquell®, Bonneuil sur Marne, France) and an aHPP system (Anios®, Lille, France) combining H_2_O_2_ with acetic and peracetic acids in MDRO environmental disinfection, and assessment of toxicity of these techniques.

## Material and methods

### Study design

This prospective cross-over study was performed during a three-month period (April through June 2012) in five medical and surgical ICUs located in the University Hospital of Lille, France. These units included three 10-bed, one 12-bed, and one 4-bed units. All rooms were single-bed. The study is in compliance with the Helsinki Declaration. In accordance with the French law, the study did not require an ethical approval. No informed consent was required by the local Institutional Review Board (CPP Nord Ouest IV) because of the non-interventional design of the study upon patients.

The primary objective was to determine the efficiency of H_2_O_2_, used after terminal cleaning, in reducing the percentage of ICU rooms contaminated with MDRO. Secondary objectives were to compare the efficiency of HPV with aHPP in reducing the percentage of ICU rooms contaminated with MDRO and to compare the residual concentration of H_2_O_2_ using these techniques.

Routine terminal cleaning was performed after patient discharge and was followed by H_2_O_2_ disinfection. During the first six-week period, two 10-bed units and the 4-bed unit were disinfected by HPV and the 22 other rooms were disinfected by aHPP. During the second six-week period, H_2_O_2_ technologies were inverted. The order of HPV, and aHPP in different units was randomized.

The French standard for the tested methods is a microbiological *in vitro* test. Both methods passed these tests. However, the current study is an *in situ* evaluation using environmental sampling.

### Environmental sampling

Twenty four microbiological samples were collected per room at three time points: just after patient discharge (T0), after terminal cleaning (T1) and after H_2_O_2_ disinfection (T2). Premoistened swabs were used to sample 5 cm^2^ of eight environmental surfaces: 1) inside the lateral part of the mattress; on highly-touched surfaces of 2) the ventilator; and 3) the monitor; 4) the underside of the overbed table; 5) on the room door handle; 6) around the sink; 7) on the keyboard for 13 computerized rooms – in storage box for other rooms; and 8) on the bedrails. In order to avoid sampling the same surface area at different time points, the sampling area was adjacent at each sampling point.

The microbiologists were blinded to H_2_O_2_ technology. Each swab was plated onto Columbia blood agar (bioMérieux, La Balme les grottes, France). An enrichment culture was made by discharging each swab into a brain heart infusion (BHI) to be re-isolated onto Columbia blood agar if positive. The plates and BHI were incubated at 37°C for 48 hours. Each bacterial colony was identified by MALDI-TOF mass spectrometry (Microflex; Bruker Daltonics, Wissembourg, France). The susceptibility of the target isolates was performed by the disk diffusion method on Mueller-Hinton agar [24].

### Standard cleaning practices

During ICU stay, the floor was cleaned three times a day using a wet sweep and once a day using a quaternary ammonium compound (Aniosurf®, Anios, Lille, France). After patient discharge, HCW cleaned and disinfected surfaces using Aniosurf®. Wipes were drenched into the bucket of quaternary ammonium solution for 15 minutes before use. Two applications were given. A five-minute contact time was observed after each application. This cleaning always followed the same sequence (from top to bottom; from cleaner to dirtier). The sink was first cleaned by a detergent (Deterg’anios®, Anios, Lille, France), rinsed with clear water and then cleaned and disinfected with Aniosurf®. After a wet sweep, floors were cleaned with Deterg’anios®, rinsed with clear water and then disinfected with sodium hypochlorite solution (contact time: 15 minutes). Before starting the study, HCW were updated concerning terminal cleaning good practices.

### HPV disinfection

After terminal cleaning, a manufacturer’s agent placed an HPV and an H_2_O_2_ catalyzer into the room. Room ventilation and door were sealed using tape. H_2_O_2_ concentration inside disinfected rooms was continuously monitored. The generator converted 30% liquid H_2_O_2_ into vapor during about 15 minutes until the dew point. After a 30-minute contact time, H_2_O_2_ was converted to oxygen and water vapor by the catalyzer. The room was opened when the inside H_2_O_2_ concentration was below 1 ppm, representing the safe permissible limit of H_2_O_2_. The time required for the entire process was approximately 1 hour 40 minutes.

### aHPP disinfection

After terminal cleaning, HCW covered screen monitors, and placed the aHPP machine in a corner of the room, powered it on, and left the room. Sixty seconds later, aerosolization of a 7% H_2_O_2_ solution associated with 0.25% peracetic acid and 30% acetic acid began for 23 minutes (suitable time for a 60 m^3^ room). After a 30-minute contact time and then two hours of room ventilation, the room was available. The time required for the entire process was approximately 2 hours 54 minutes.

### Measurement of H_2_O_2_ concentration

H_2_O_2_ concentration was measured at the end of vaporization/aerosolization in the corridor and rooms next to the treated room and in the treated room at the end of the entire process. H_2_O_2_ concentration was recorded by two methods: an electronic one (Pac III®, Dräger) and a chemical one (Dräger tubes® and Accuro® pump, Dräger, Pittsburgh, PA, USA). For aHPP, acetic acid concentration was analyzed using a chemical process (Dräger tubes® and Accuro® pump, Dräger).

### Clinical data

The characteristics of the room occupants were collected, including MDRO status and ICU-length of stay. MDRO were defined as MRSA, *P. aeruginosa* resistant to ceftazidime or imipenem, extended spectrum β–lactamase (ESBL)-producing Gram-negative bacilli (GNB), imipenem resistant *Acinetobacter baumannii* (IRAB) and VRE. During the study period, all ICU patients were screened (nasal and anal swabs) for MDRO at ICU admission and once a week.

### Statistical analyses

SAS software (9.3 version, SAS Institute Inc., Cary, NC 27513, USA) was used for data analysis. Based on the prevalence of 30% to 40% of MDRO in our ICU, we estimated an incidence of rooms contaminated with MDRO after routine terminal cleaning (T1) of 20% and after H_2_O_2_ treatment (T2) of 5%. Studying 76 rooms in each group (aHPP and HPV) would allow detection of this difference with an 80% power and a two-tailed significance level of 0.05.

Results are presented as frequency (percentage) for categorical variables and median (interquartile range) for quantitative variables. The normality of distribution was tested by a Shapiro Wilk test. To compare groups at different time points (T0, T1, T2), the chi-squared test or Fisher’s exact test, and the Mann–Whitney U-test were used for qualitative and quantitative variables, respectively. All *P* values were two-tailed. The statistical significance was defined as *P* <0.05.

Comparisons between T0 and T1, and T1 and T2 were performed using McNemar’s test. In order to identify rooms at higher risk for positivity for MDRO, rooms were classified based on occupant status regarding MDRO, and duration of ICU stay ≥8 days (median length of ICU stay in study population).

## Results

**Figure 1:**
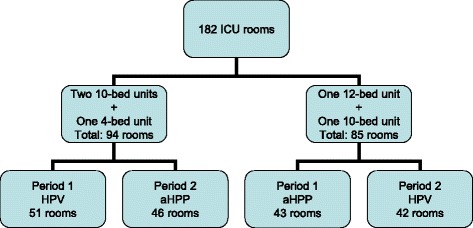
**Study flowchart.** HPV, hydrogen peroxide vaporization; aHPP, aersolization of hydrogen peroxide.

One hundred and eighty two rooms were studied, including 93 (51%) disinfected with HPV and 89 (49%) with the aHPP system (Figure 1). Occupancy rate was 90%.

### Routine terminal cleaning and H_2_O_2_ efficiency

At T0, 141 out of 182 (77%) rooms were contaminated with at least 1 bacterium and 15 (8%) with at least 1 MDRO (Table 1). Routine terminal cleaning was associated with a significant reduction of bacterial environmental contamination (*P* <0.001). However, no significant difference was found in the percentage of MDRO contaminated rooms between T0 and T1. The percentage of rooms contaminated with bacteria or with MDRO was significantly lower at T2 compared with T1.

**Table 1:** **Efficiency of terminal cleaning and H**_**2**_ **O**_**2**_ **disinfection**

	**T0 number = 182**	**T1 number = 182**	**∆ T0-T1**	***P***	**T2 number = 182**	**∆ T1-T2**	***P***
Rooms contaminated with at least one bacterium	141 (77)	70 (38)	- 39%	<0.001	10 (5)	- 33%	<0.001
Rooms contaminated with at least one MDRO	15 (8)	11 (6)	- 2%	0.371	1 (0.5)	- 5.5%	0.004

Data are numbers (%). MDRO, multidrug-resistant organism.

At T0, MDRO were mainly located near the sink. Results on the efficiency of terminal cleaning and H_2_O_2_ disinfection in reducing MDRO contamination of different sites are presented in Table 2. At T0, ESBL-GNB were the most frequently identified MDRO (50%) followed by IRAB (29%), MRSA (17%) and MDR *P. aeruginosa* (4%). Only one MDRO was identified per room at T0, except for one room where two different ESBL-GNB were found. At T1, four of the fourteen isolated MDRO were not identified at T0.

**Table 2:** **MDRO contamination of different environmental sites at different time points**

**Rooms contaminated with at least one MDRO on:**	**T0 number = 182**	**T1 number = 182**	**T2 number = 182**
Mattress	1 (0.5)	1 (0.5)	0 (0)
Ventilator	3 (2)	0 (0)	0 (0)
Monitor	4 (2)	0 (0)	0 (0)
Overbed table	0 (0)	0 (0)	0 (0)
Room door handle	3 (2)	0 (0)	0 (0)
Sink	9 (5)	9 (5)	0 (0)
Keyboard (58 rooms)	0 (0)	0 (0)	0 (0)
Storage box (124 rooms)	0 (0)	1 (0.8)	1 (0.8)
Bedrails	3 (2)	1 (0.5)	0 (0)

Data are numbers (%). MDRO, multidrug-resistant organisms.

The percentage of microbiological samples positive for MDRO was significantly lower at T1, compared with T0 and at T2, compared with T1. The percentage of microbiological samples positive for ESBL was significantly lower at T2, compared with T1. No significant difference was found in the rate of samples positive for other MDRO between T2 and T1 (Table 3).

**Table 3:** **Type of microorganisms identified on room surfaces**

**Number of microbiological samples**	**T0 number = 1456**	**T1 number = 1456**	**T2 number = 1456**
MDRO	23 (1.5)	14 (0.96)*	2 (0.13)*
ESBL	12 (0.82)	14 (0.96)	2 (0.13)*
MRSA	4 (0.27)	0 (0)	0 (0)
IRAB	6 (0.41)	0 (0)	0 (0)
Resistant *P. aeruginosa*	1 (0)	0 (0)	0 (0)

Data are numbers (%). ESBL, extended spectrum β-lactamase producing Gram negative bacilli; IRAB, impipenem resistant *Acinetobacter baumannii*; MDRO, multidrug-resistant organisms; MRSA, methicillin resistant *Staphylococcus aureus*. *P = 0.004 versus T0, <0.001 versus T1, <0.001 versus T1, respectively. **P** >0.2 for all other comparisons.

### Comparison of H_2_O_2_ technologies

The percentage of ICU rooms contaminated with MDRO at T2 was similar in the HPV group compared with the aHPP group (1 out of 51 (1.9%) versus 0 out of 49 (0%), *P* = 0.313). Before H_2_O_2_ disinfection, bacterial and MDRO environmental contaminations were similar in the two groups.

### Patient characteristics

Seventy four out of 177 (42%) room occupants (5 missing data) were colonized or infected with MDRO, including 43 (24%) ESBL-GNB, 18 (10%) MDR *P. aeruginosa*, 15 (8%) MRSA and 11 (6%) IR*A*B. No VRE was identified during the study period. Only one patient suffered from *Clostridium difficile*-associated disease. At ICU admission, MDRO were identified in 27 (15.2%) patients, including 10 (5.6%) ESBL, 8 (4.5%) *P. aeruginosa*, 6 (3.3%) MRSA and 3 (1.6%) IRAB.

Median ICU length of stay was 8 days (4, 18). ICU length of stay was significantly longer in rooms contaminated with MDRO compared with those not contaminated with MDRO (23 (15, 35) days versus 7 (4, 15) days, *P* = 0.003). In rooms contaminated with MDRO at T0, occupants were known as MDRO carriers in 10 out of 15 (67%) cases compared with 5 out of 162 (3%) in rooms where occupants were not colonized or infected with MDRO, *P* <0.005.

The percentage of patients with MDRO was similar in rooms disinfected using aHPP and those disinfected using HPV (38/89 (44%) versus 36/93 (40%), respectively, *P* = 0.731). The percentages of different MDRO were also comparable in the two groups. ICU length of stay was similar in aHPP and HPV groups (8 (4, 15) days versus 8 (4, 18) days, respectively, *P* = 0.975).

### Classification of ICU rooms based on patient MDRO status and length of ICU stay

**Figure 2:**
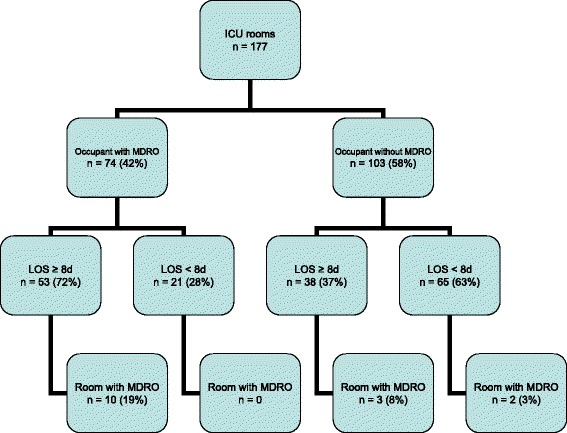
**Classification of study rooms based on patient characteristics.** LOS, length of stay; MDRO, multidrug-resistant organisms.

The percentage of rooms contaminated with MDRO was significantly higher in rooms with length of ICU stay ≥8 days occupied by a patient with MDRO compared with rooms with length of ICU stay <8 days where the prior room occupant was not an MDRO carrier (10 out of 53 (19%) versus 2 out of 65 (3%), *P* = 0.012, odds ratio (OR) (95% confidence interval (CI)) 7.3 (1.5, 35.1)) (Figure 2).

### Toxicity

Four toxicity tests were performed in aHPP rooms and five in HPV rooms. H_2_O_2_ and acetic acid were never found in the corridor or in the rooms next to the studied room during the process. At the end of the HPV process, H_2_O_2_ concentrations inside tested rooms were between 0.4 and 0.7 ppm. At the end of aHPP disinfection, the H_2_O_2_ rate ranged from 0.5 to >3 ppm inside tested rooms; acetic acid was <5 ppm. Persons who entered aHPP treated rooms described an unpleasant smell and irritation of the eyes and upper airways.

## Discussion

Our results suggest that routine terminal cleaning followed by H_2_O_2_ treatment is more efficient than routine terminal cleaning alone for disinfection of MDRO contaminated ICU-rooms after patient discharge. No significant difference was found between aHPP and HPV regarding percentage of ICU rooms contaminated with MDRO after terminal cleaning and disinfection using these techniques. The residual concentration of H_2_O_2_ appears to be higher using aHPP compared with HPV.

Our study demonstrates a significant reduction in the percentage of MDRO contaminated rooms using H_2_O_2_ techniques. The strength of this study is the large number of sequential environmental samples performed to determine the efficiency of these techniques. Previous studies demonstrated that HPV was an efficient technique to improve environmental disinfection after patient discharge [13-15,17-23]. This efficiency has been demonstrated *in vitro* and *in vivo* during endemic and epidemic periods. However, several limitations of these studies should be outlined, including *in vitro* design, small number of studied ICU rooms, absence of systematic environmental samples and focus on specific MDRO or specific population. A recent observational clinical study found environmental decontamination with HPV to be associated with significantly reduced risk for patient acquisition of MDRO [16]. While the number of sampled rooms was high (n = 1,039), environmental samples were only performed at one time-point in a small proportion of studied rooms (11.7%). In addition, neither rooms nor units were randomly assigned to the intervention.

Our study is the first to assess the efficiency of an aHPP system using a solution containing H_2_O_2_ and acetic and peracetic acids, and to compare it with HPV. Several studies demonstrated the *in vitro* and *in situ* effectiveness of silver-based aHP in disinfecting inanimate surfaces. The bacterial load reduction was incomplete and has been proven for MRSA, VRE, *A. baumannii*, *C. difficile* and *geobacillus stearothermophilus* biological indicators [25-31]. However, the conclusions of these studies could not be applied to the aHPP technique using acetic and peracetic acids. Two previous studies compared HPV to an aHP treatment combining H_2_O_2_ with silver cations [32,33]. Although these *in vitro* experiments highlighted a greater reduction of bacterial load with HPV, our study found similar efficiency of HPV and aHPP. These results suggest that aHPP might be more efficient than aHP. However, further studies directly comparing these techniques are required to confirm this hypothesis.

Terminal cleaning in France is probably different from that performed in the USA or other parts of the world. The major part of MDRO was isolated around the sink, suggesting that cleaning of this area should be improved. This improvement could be sufficient to reduce vertical transmission of MDRO via room surfaces. However, previous studies have clearly shown that improvement in terminal cleaning was not sufficient to control MDRO transmission via surfaces [12]. H_2_O_2_ and peracetic acid are powerful oxidants with bactericidal, fungicidal, sporicidal and virucidal effects. However, H_2_O_2_, acetic and peracetic acids are corrosive and caustic, and are toxic to human beings at high doses (>1 ppm, >10 ppm and >0.17 ppm, respectively). Governments impose occupational exposure limits to chemical products. The H_2_O_2_ long-term exposure limit is 1 ppm/8 hours in several countries (France, USA, UK). Our results suggest that residual concentrations of H_2_O_2_ are higher using aHPP compared with HPV. However, the small number of tests performed to determine these concentrations preclude definite conclusions regarding the toxicity of aHPP. In addition, in the absence of data concerning peracetic acid concentration, we cannot affirm the safety of aHPP system.

In practice, H_2_O_2_ decontamination devices are associated with a longer waiting time between two subsequent admissions in the same room, approximately 1 hour 40 minutes for HPV and 3 hours for aHPP. They are also associated with increased hospital costs. One could argue that these costs are counterbalanced by lower costs related to ICU-acquired infections management. However, cost-effectiveness analyses are required to confirm this hypothesis. In our experience, no alteration of medical devices was observed. The Environmental Protection Agency (USA) has reported a medium-term compatibility of HPV with various materials and electronic equipment [34].

In spite of a high rate of patients with MDRO (42%), the percentage of ICU rooms contaminated with MDRO at patient discharge was relatively low (8%). However, this rate is in line with previously reported results [17,21]. Three potential explanations could be given for this result. First, the relatively short median length of ICU stay (eight days) did not allow heavy contamination of the environment with MDRO. Second, bacteriological samples performed at patient discharge might have missed the contaminated surfaces. However, eight swabs were performed per room at T0, allowing examination of the most frequently touched surfaces by the patient and HCW. Third, our strict terminal cleaning protocol, including the routine use of sodium hypochlorite solution might have contributed to this result. However, it is unlikely that floor cleaning had an impact on the prevalence of MDRO contaminated rooms because all sampled areas were high touched surfaces unconnected to the floor.

ICU rooms at the highest risk for contamination with MDRO were those where patients stayed for a long period of time (≥8 days), and where the prior room occupant was an MDRO carrier. This might be helpful to apply a targeted strategy for disinfection of ICU rooms using H_2_O_2_ techniques only in these at high-risk rooms. However, further studies are needed to evaluate such a strategy.

Our study has some limitations. First, the number of rooms contaminated with MDRO was relatively small. As a consequence, no definite conclusion could be drawn on the comparison of the efficiency of different H_2_O_2_ generators in MDRO environmental disinfection. However, this comparison was a secondary outcome. Second, it is important to highlight that the H_2_O_2_ generators used different approaches and different chemical compositions (30% of H_2_O_2_ for HPV versus 7% of H_2_O_2_, 30% of acetic acid, and 0.25% of peracetic acid for aHPP). Third, no definite conclusion could be drawn on the efficiency of H_2_O_2_ decontamination on different types of MDRO. A recent study [35] suggested that the reduction of a commercially available biological indicator cannot always be extrapolated to other microorganisms, especially MRSA. The production of catalase, which could break down the H_2_O_2_, might result in a reduction of the effectiveness of these techniques. However, another recent study suggested that HPV achieved a 6-log reduction, whereas aHP generally achieved less than a 4-log reduction on the biological indicators and in-house prepared test discs containing approximately 10^6^ MRSA, *C. difficile* and *A. baumannii* [33]. Fourth, this study is merely environmental and the impact of H_2_O_2_ decontamination on the incidence of MDRO colonization or infection was not studied. Finally, our study was conducted in a single institution. Therefore, our results may not be generalizable to other institutions with different infection control practices and rates of MDRO.

## Conclusions

Routine terminal cleaning followed by H_2_O_2_ treatment is more efficient than routine terminal cleaning alone for disinfection of MDRO contaminated rooms in the ICU. No significant difference was found between aHPP and HPV regarding efficiency in disinfection of MDRO contaminated rooms. Further studies are needed to evaluate the toxicity of aHPP techniques.

## Key messages

- Hydrogen peroxide techniques are efficient in disinfecting ICU rooms contaminated with MDRO.
- No significant difference was found between aHPP and HPV regarding their disinfection efficiency.
- Further studies are needed to evaluate the toxicity of aHPP.

## Abbreviations

aHP: aerosolization of hydrogen peroxide
aHPP: aerosolization of hydrogen peroxide and peracetic acid
ESBL: extended spectrum betalactamase producing
GNB: gram negative bacilli
H_2_O_2_: hydrogen peroxide
HCW: healthcare workers
HPV: hydrogen peroxide vaporization
ICU: intensive care unit
IRAB: imipenem resistant *Acinetobacter baumannii*
MDRO: multidrug resistant organism
MRSA: methicillin resistant Staphylococcus
VRE: vancomycin resistant enterococcus
